# Eco-Design of Polymer Matrix Composite Parts: A Review

**DOI:** 10.3390/polym15173634

**Published:** 2023-09-02

**Authors:** Sergiu Lazăr, Dan Dobrotă, Radu-Eugen Breaz, Sever-Gabriel Racz

**Affiliations:** Faculty of Engineering, Lucian Blaga University of Sibiu, 550024 Sibiu, Romania; sergiu.lazar@ulbsibiu.ro (S.L.); dan.dobrota@ulbsibiu.ro (D.D.); gabriel.racz@ulbsibiu.ro (S.-G.R.)

**Keywords:** eco-design, composite materials, eco-innovation, sustainable design

## Abstract

This research presents a series of analyses related to the eco-design of polymer matrix composite parts, addressing various aspects of it. The main objective was to clarify the definition of ecological design, the benefits of its implementation and its importance in all stages of obtaining a product (design, manufacturing, recycling). Global environmental issues are presented, emphasizing the importance of adopting sustainable approaches in product design and manufacturing. Special attention is paid to the analysis of waste recycling technologies for polymer matrix composite materials. The analysis carried out identifies specific ecological design principles applicable to these materials and presents recent trends in the field. Relevant case studies are highlighted, demonstrating the benefits of ecological design in order to obtain sustainable products. Additionally, the conducted research allowed for finding answers to the questions “what”, “why”, “when” and “how” it is necessary to apply the principles of eco-design in the case of composite materials with a polymer matrix. In general, the research promotes eco-design as an indispensable strategy for sustainable and responsible production, inspiring companies to adopt these principles for the benefit of the environment and their business performance.

## 1. Introduction

In recent decades, polymer matrix materials have been well developed and used in wide applications (biomedicine, automotive industry, construction materials, etc.), all of which involve high consumption of raw materials and energy. Also, irresponsible extraction of raw materials, as well as processing of raw materials, account for over 90% of the issues regarding biodiversity loss [1]. Moreover, the growth and development of industrial activities both at the European Union level and worldwide have detrimental consequences for the environment and population. The world’s largest polluters, including China, the USA, and India, have strengthened their position as industrial leaders, experiencing annual growth rates of up to 35% [2].

The extraction and processing of raw materials, along with the exploitation of all planetary resources, have numerous negative effects on the planet [3]. The most well-known effects experienced are global warming and its consequent desertification. One of the immediate effects is desertification, which refers to the degradation of soil and water resources in dry and semiarid areas, leading to biodiversity and soil fertility loss, thereby affecting the capacity to support human life and ecosystems. According to the United Nations (ONU), approximately 24% of the Earth’s land surface was already affected by soil degradation by the end of the 2010–2020 decade, and desertification is currently occurring in over 100 countries. This process is caused by several factors, including climate change, excessive soil use, deforestation, and population growth. Among the solutions proposed by ONU experts analyzing this phenomenon are the reduction of pollutant emissions and greenhouse gases, the reduction of energy and water consumption in industrial activities, rational exploitation of planetary resources, and the reduction or cessation of deforestation [4,5]. However, to slow down or mitigate this process, actions must be taken regarding global warming since the warmest ten years in the history of meteorological measurements were recorded in the period 2010–2022 [6].

Furthermore, when looking at composite materials with a polymer matrix, which are integral parts of many equipment structures, it has been found that there are different estimates regarding their utilization percentage in construction. For example, some consider that in the case of a 1163 kg piece of equipment, approximately 150 kg represents composite materials with a polymer matrix, which is approximately 12.9% [7]. In general, composite materials with a polymer matrix are increasingly used in various components of modern automobiles, including the body, seats, upholstery, electrical and electronic components, and many others [7]. Consequently, the proportion of composite materials used in the construction of a medium-sized car can vary between 10 and 50%, depending on several factors such as material costs, vehicle performance requirements, availability of manufacturing processes, or technological advances [8]. It is important to note that as car manufacturers aim to reduce weight and improve efficiency, the use of lightweight composite materials is becoming more common [7]. Other researchers consider the recycling of composite waste materials as a global hazard [9]. Moreover, it is currently necessary to pay increased attention to the development of durable and recyclable composite materials that can reduce the carbon footprint and contribute to a more sustainable environment. However, as stated by several researchers, the recycling of composite materials with a polymer matrix is still challenging [10]. Theoretically, there are several recycling solutions, but they only artificially prolong the life of a polymer molecule and are less useful. For example, a composite material with a polymer matrix can be regranulated, compounded (with virgin material), or only reinforced with glass fiber [11]. However, due to the recycling processes that involve additional heating and cooling, the base material undergoes molecular changes, and the quality of the polymer will be affected [12]. Therefore, often the polymer obtained from recycling waste is used in various asphalt mixtures or reintegrated into the injection molding process with difficulty [13,14,15,16].

Considering this imbalance caused by the depletion of certain raw material sources and the growth of industrial activities in polluting countries, as well as the challenges in recycling large quantities of composite materials with a polymer matrix, sustainable alternatives must be urgently found. These alternatives should address ecological, economic, and social aspects [17]. In this regard, it is imperative to conduct an analysis to identify the most important factors that contribute to greenhouse gas emissions or pollutants, factors that increase energy consumption, factors that affect the health and safety of operators or the areas they live in, and economic factors that increase production costs. An integral part of this analysis is the concept of eco-design, which is often proposed by researchers and engineers under various forms such as eco-innovation, design for the environment, design for sustainability, ecological design, green design, and others.

Eco-design refers to the concept that imposes rigorous or demanding conditions regarding the life cycle of a product in terms of its economic, ecological, and social impact [18]. The main goal of this approach is to reduce pollutant emissions, slow down climate change, comply with regulations imposed by the European Commission [19], and align the product development process with the aspirations of the United Nations’ Agenda for Sustainable Development [5].

The history of eco-design can be traced back over a long period, starting in the 1920s when architect and designer Richard Buckminster Fuller began creating plans for structures, machines, and other objects that promote responsible resource consumption [20]. Over the decades, eco-design has evolved and become a design and production method that considers the environmental impact at all stages of product development. The term “eco-design” was first mentioned in a 1996 book by Sim van der Ryn and Stewart Cowan, where the authors argued for the seamless integration of human activities with natural processes to minimize the destructive impact on the environment. Today, eco-design is an important part of sustainable development and continues to evolve to address increasingly significant environmental challenges [21].

Furthermore, in addition to these attempts to improve existing production technologies that primarily rely on the mechanical design of components, legislative improvements on a global scale will be necessary. The research theme has a global impact. The development of legislative packages to facilitate the circular economy must go hand in hand with the development of less-polluting technologies. Education and professional training need to align with the principles of sustainability so that the skills of the workforce facilitate the implementation of eco-design but without being limited to this trend [18,21].

The eco-design of parts made of composite material with a polymer matrix must also take into account the interfacial adhesion between nanoparticles and polymer matrices, because the polymer chains that form such composites significantly influence their properties. Also, by applying eco-design principles in a developed way in the case of cellulose- and carbon-based nanocomposition, it allows to create their mechanical properties but also the homogeneity of such materials [22]. Moreover, the application of the eco-design principles can significantly improve the properties (thread extraction force, energy consumed by the interface, static and kinetic friction interface) but also increase the number of potential applications, especially in areas such as obtaining protective sports-clothing equipment but also in the field of automobile construction [23].

Therefore, the main objective of the conducted research was to analyze research articles that address topics related to geometric, dimensional, and structural optimizations of various categories of products made from composite polymer materials. These optimizations should have a positive impact on the product life cycle, providing benefits in terms of technological and energy consumption, raw material exploitation, behavior under different mechanical stresses or chemical reactions, life span, repair/servicing possibilities, and recyclability.

Thus, the article aims to identify existing research gaps in the field of eco-design for composite materials while proposing new directions to generate eco-design for products made from composite materials with a polymer matrix. It is important to note that the purpose of this work is to analyze the results of research presented in scientific papers published in recent years, focusing on eco-design on the one hand and identifying trends in the development of new composite materials on the other hand, to align them with economic, ecological, and social requirements.

Taking into account all the aspects mentioned above, the article will attempt to provide an overview of eco-design for composite materials with a polymer matrix by analyzing design, manufacturing, end-of-life considerations, and material reintegration into the production cycle in various forms.

## 2. Research Questions in Eco-Design

Although the first mention of the concept of eco-design dates back to the 1920s, even now, over 100 years later, there are numerous discussions and debates regarding:

- what it represents and how eco-design should be implemented on an industrial scale;
- why it needs to be swiftly implemented;
- when eco-design should be addressed to ensure optimization of product development processes.

Furthermore, since the main objective of every business operator is to maximize profits, it can be hypothesized that eco-design will reduce production costs, possibly with a minimal initial investment that will quickly amortize.

Based on the aforementioned shortcomings expressed in the industry through the lack of response in implementing this concept, the paper aims to clarify the most important aspects regarding how eco-design should be understood. At the same time, solid arguments will be provided to demonstrate the hypothesis that eco-design will generate financial, ecological, and social benefits for all parties involved, creating a win–win relationship for producers, customers, and society. To materialize this desire, the article will extensively address the questions of “**what**”, “**why**”, “**how**”, and “**when**” of the eco-design.

**Q1—What** is eco-design of composite materials and **why** is it necessary to implement it in the design and manufacturing stages?
**Q2—How** does eco-design relate to the selection of polymer composite materials?
**Q3—When** can it be said that a product made from polymer composite materials meets the requirements of eco-design?

## 3. Literature Review Methodology

The answer to the above questions and the establishment of the hypotheses were achieved through the analysis of scientific literature databases, synthesis of the most important information from relevant articles, information classification, and result analysis.

To synthesize the most important information, an analysis of various databases such as Web of Science, Scopus, Ebsco, Springer, Elsevier, etc., was conducted. Search engines such as Google Scholar, JSTOR, Web of Science, Wiley Online Library, ACM Digital Library, and Scopus were used to identify articles of interest. Additionally, two artificial intelligence platforms dedicated to scientific research, Consensus and cite, were utilized to facilitate the identification of relevant articles for the research topic. The synthesis of information was conducted in a manner that allowed for the generation of arguments or counterarguments to the analyzed hypotheses and provided a more comprehensive understanding of eco-design.

**Figure 1:**
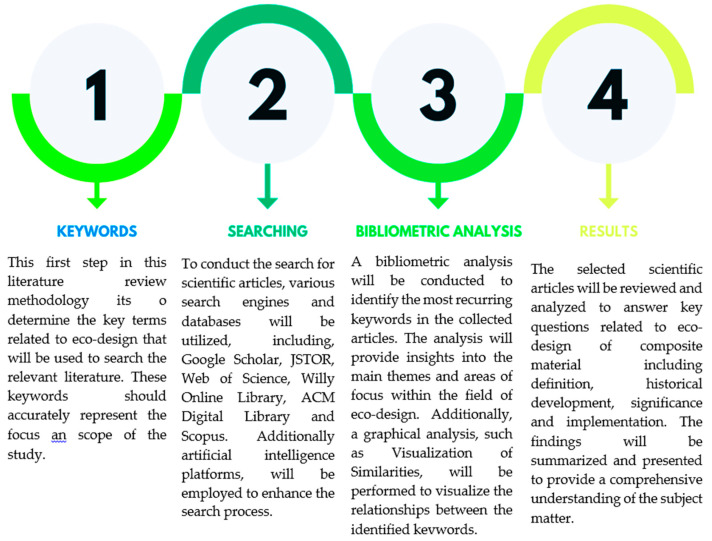
Literature review methodology.

The analysis of eco-design of polymer composite materials was further expanded through the application of term classification, using a visual representation known as VoS (Visualization of Similarities), as shown in Figure 1. VoS is a method of visualizing scientific data that is based on the principle that information is easier to understand and interpret when presented graphically. VoS involves representing data in the form of diagrams that illustrate the relationships between different elements, such as scientific articles, researchers, or institutions. These diagrams are used to illustrate how the elements are interconnected and to provide an overview of the structure and characteristics of the data. This method is employed to offer an overview of the data and to identify patterns, trends, and relationships within scientific data.

The final part of the research encompasses the results and discussions. Furthermore, through the conducted research, efforts were made to obtain answers that would help verify the validity of the hypotheses.

As previously stated, by using search engines such as Google Scholar, JSTOR, Web of Science, Wiley Online Library, ACM Digital Library, and Scopus, as well as artificial intelligence platforms, extensive studies and scientific papers related to the concept of eco-design of polymer composite materials were found. The search was conducted using the following keywords: eco-design, sustainable design, design for environment, ecological design, green product design, design for sustainability, eco-design of composite materials, recycling of composite materials, increasing life span of composite materials, innovative design for composite materials.

Simultaneously, scientific articles providing ecological solutions for polymer composite materials were identified using keywords such as eco-friendly composite materials, new composite materials, green composite materials, bio composite materials, natural fiber composite materials, innovative composite materials, sustainable composite materials. Moreover, articles focusing on the recycling of polymer composite materials constituted another area that was carefully explored, using terms such as composite recycling, polymers recycling, thermosets recycling, polymers waste management.

The analysis of information regarding the eco-design of composite materials encompassed important aspects discussed in relevant studies and scientific articles published in recent years. Through the conducted research, the aim was to provide the latest insights into how industry and researchers perceive eco-design. Thus, a comprehensive analysis of the state of the art until 2023 was obtained.

Another important aspect of the literature review involved a detailed analysis of innovative composite materials. In this stage, various types of polymer composite materials were analyzed, evaluating their sustainability, including their environmental impact and end-of-life management options. By comparison, current trends in composite materials were addressed to identify the main differences in terms of the three sustainability dimensions: environmental, economic, and social.

Furthermore, by studying the specialized literature, the most important information regarding the evaluation of the life cycle of products made from polymer composite materials was extracted. Specifically, this section analyzed the impact of polymer composite materials on ecosystems throughout their life cycle, from raw material extraction to manufacturing, use, and disposal. It also covered key points in the manufacturing process, such as the energy consumption and environmental impact of manufacturing processes used to produce components from polymer composite materials, identifying opportunities for optimization.

Because recycling is one of the important aspects of eco-design, a series of valuable scientific papers addressing the topic of composite material recycling, especially “green” composites, and their impact on sustainability were studied.

In conclusion, this research will fully or partially answer all the questions in the hypotheses and provide clear statements regarding all aspects of eco-design of composite materials, based on the existing scientific background up to this point.

For a better understanding of the evolution and structure of eco-design, the Visualization of Similarities (VoS) technique was used, which graphically represents the relationship between multiple elements and keywords from the analyzed scientific papers. The main purpose of choosing this technique was to provide an overview of eco-design and polymer composite materials, which can help identify patterns, trends, and interrelationships between the two major subjects.

**Figure 2:**
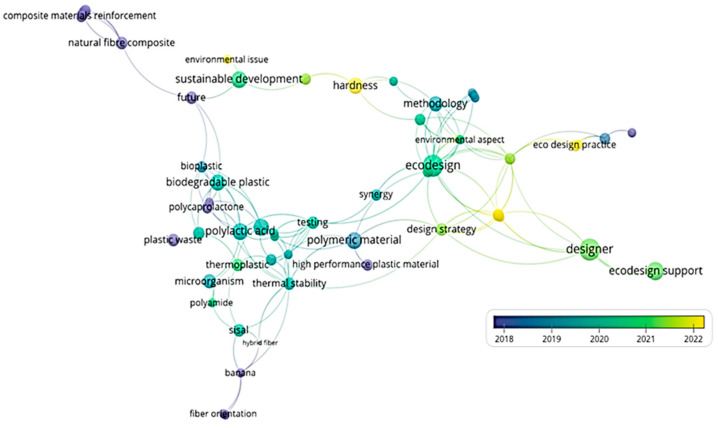
Bibliometric analysis for composite material with polymeric matrix eco-design.

Two types of VoS visualizations were used: cloud of words and map of science. Through the map of science, Figure 2 provides a comprehensive representation of the correlations that can be established between specific topics and/or domains, focusing on the intersection of eco-design and composite materials. Figure 2 also shows the evolution over time of topics and relationships between terms from 2018 to 2022. 

To accomplish this section of the research methodology, the VOSviewer version 1.6.19 platform provided by the Centre for Science and Technology Studies, Leiden University, The Netherlands, was used. It is available at https://www.vosviewer.com/ accessed on 10 July 2023.

## 4. Evaluation of Questions Q1, Q2, and Q3 for the Eco-Design of Polymer Matrix Composite Parts

**Q1. What is eco-design of composite materials, and why is its implementation necessary in the design and manufacturing stages?**

In an attempt to provide the most up-to-date definition of eco-design, we had to start with the description of sustainability as the capacity of the biosphere to coexist with human civilization, since sustainability is closely related to both the purpose and the means/methods of eco-design [5,21]. If we analyze the definition of eco-design in articles published in recent years, we observe that each work presents only a part of the concept without synthesizing the ecological, economic, and social role of its implementation in the production cycle.

For example, in scientific papers whose purpose is to highlight the existence of alternative reinforcement materials (natural fibers), eco-design is viewed solely from the perspective of the numerous ecological advantages it brings. At the same time, articles that focus on the benefits of shortening supply chains as part of eco-design only analyze the economic sphere of these improvements. It is extremely rare for authors to analyze both aspects of sustainability together or to even introduce the third axis into the discussion—the social axis. A much more practical example of what was mentioned above is provided by the European Environment Agency, which defines eco-design in its glossary [19] as the integration of environmental aspects into product development processes by balancing ecological and economic requirements. Ecological design considers environmental aspects in all stages of product development, aiming for products that have the smallest possible impact on the environment throughout their life cycle.

Such an approach is not considered incorrect, but based on the industry’s understanding of eco-design, we can demonstrate that this definition is incomplete. A significant argument arises from analyzing the multiple definitions of eco-design under its various names and concepts, observing that numerous recurring terms are encompassed within this field in various scientific works, such as [5,12,17,18,21,24]. These recurring terms can be classified into four main categories:

- sustainability and ecological performance;
- energy efficiency and natural resources;
- sustainable raw materials;
- technological innovation and standards.

**Figure 3:**
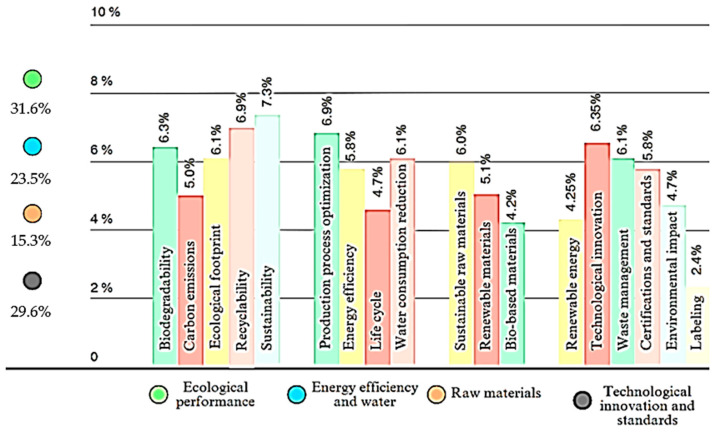
Recurrent terms in the definition of eco-design.

This classification allows for a deeper understanding of the relevant domains and priorities within the eco-design of composite materials with a polymer matrix. In order to highlight the contribution and importance of the most frequently encountered 18 words in the definitions of the eco-design concept, we have developed an explanatory figure, Figure 3. This figure aims to illustrate the interconnections and relationships between these key terms, providing a visual perspective on their complexity and interdependence within the eco-design of composite materials with a polymer matrix.

By exploring and understanding these terms and categories in depth, we can contribute to the development and implementation of innovative and sustainable solutions in the field of composite materials. This eco-design-oriented approach not only optimizes the technical performance of products but also has a positive impact on the environment throughout their life cycle.

Through careful analysis of these terms, we have observed that sustainability is the most frequently encountered and dominant term in the definition of eco-design for composite materials with a polymer matrix. This concept encompasses not only ecological aspects but also economic and social ones, being essential in promoting sustainable and responsible practices in all phases of the materials’ life cycle. Optimizing extraction, manufacturing, and transportation processes represent another key component of eco-design, directly impacting efficiency and reducing environmental impact [18].

Furthermore, recycling and product repair are crucial elements in eco-design, contributing to responsible waste management and extending the life span of products in a circular economy approach [20]. However, a less commonly used term, but with significant potential, is labeling. Energy labeling or labeling related to the structure of the polymer composite can provide essential information to consumers and manufacturers, helping them make informed decisions and promoting environmentally friendly composite materials. In this context, the structural optimization of materials can play an essential role in facilitating the recycling process and achieving superior sustainability performance.

Therefore, understanding and properly applying these key terms in eco-design for composite materials with a polymer matrix are essential for the development of innovative and sustainable solutions, aiming to protect the environment and promote a circular economy. Moreover, the eco-design approach can contribute to reducing production costs through optimizing technological consumption and creating shorter supply chains [20].

A primary argument supporting this statement is provided by scientific articles that focus on eco-design from the perspective of shortening supply chains [25]. One of the conclusions of an article [25] is that by improving the supply chain through shorter distances from extraction to production, two main effects can be achieved: reducing logistical costs and integrating the population near the extraction area into industrial production activities. Consequently, there will be more and more diverse job opportunities in the extraction area, leading to local economic growth and significantly improving the social aspect of sustainability [26]. Furthermore, there are ecological benefits: logistic energy consumption will be reduced as the transportation of raw materials from extraction to production will cover a significantly shorter distance. Considering that 58.7% of global electricity generation is produced by burning fossil fuels, consuming fewer fossil fuels for commuting to and from work will have a positive environmental impact [26,27].

Therefore, based on the analyzed scientific articles and understanding how the industry perceives eco-design, we can state that the most current and comprehensive definition of eco-design is as follows:

Eco-design of composite materials is an approach used in product design that focuses on the production, development, use, and end of life (EOL) of composite materials, taking into account the technical performance of products, their environmental impact throughout their life cycle, and reducing production costs through minimizing costs and technological consumption and shortening supply chains.

The attributes of implementing eco-design in the production cycle include reducing the amount of waste generated during production, use, and disposal of the product, using recyclable materials and sustainable energy, employing low greenhouse gas emission production technologies, and reducing water consumption in extraction and production [24,25].

Implementing this sustainable approach within a company that produces components made from composite materials with a polymer matrix can bring numerous benefits. By applying eco-design strategies, the company can reduce its environmental impact, increase energy efficiency, and improve product quality [24,28]. As a result, the company can benefit from several advantages, which will be further detailed.

Firstly, implementing eco-design can help reduce costs. Such a strategy can identify cheaper and more durable materials, minimize waste, and optimize production. By saving resources and reducing production costs, the company can achieve higher profitability and greater competitiveness compared to competitors [21].

Moreover, eco-design can contribute to improving product quality. By developing more efficient designs, the company can create more reliable and durable products with longer life spans and better performance [29]. Thus, eco-design can aid in creating high-quality products, and by committing to producing more sustainable products and demonstrating concern for environmental protection, the company can attract customers interested in more sustainable products. Additionally, using more durable and less-polluting materials can help build a strong brand image, which can contribute to increasing customer loyalty [30].

Furthermore, eco-design can help comply with environmental regulations and protections [31]. By reducing energy and resource consumption and minimizing waste, the company can more easily adhere to environmental norms and avoid fines and other penalties [32]. Additionally, implementing eco-design can help reduce greenhouse gas emissions and other pollutants, thereby contributing to environmental preservation [29].

Lastly, eco-design can contribute to overall sustainability. By using durable and less-polluting materials, the company can reduce its environmental impact and contribute to sustainable business development [33]. Thus, implementing eco-design can aid in increasing overall sustainability, protecting the environment, and achieving sustainable business growth. Implementing eco-design can help a company reduce its impact on the environment by reducing the consumption of natural resources, greenhouse gas emissions, and waste generation [34].

**Q2. How does eco-design relate to the selection of polymer composite materials?**

All industrial sectors are constantly searching for composite materials due to their numerous advantages: high strength and stiffness with reduced mass [35]. The analyzed scientific studies show that the industry is particularly interested in new characteristics of composite materials, such as regenerability, reduced environmental impact, and cost. Consequently, there is an increasing focus on research and innovation in composite materials reinforced with natural fibers due to their lower acquisition cost and significantly reduced negative impact on the environment compared to composite materials with a polymer matrix reinforced with synthetic fibers (carbon, glass, etc.) [28,36,37,38].

More specifically, during the literature review, two ecological trends were identified regarding polymer matrix composite materials: the use of biodegradable polymer matrix composite materials and the use of polymer matrix composite materials reinforced with natural or hybrid fibers.

**Q2.1. Biodegradable polymer composite materials**

Biopolymers are polymers produced from natural sources such as plants, animals, and microorganisms. These polymers are biodegradable and recyclable, making them a more sustainable alternative to traditional polymers derived from petrochemical sources. Biopolymers can be used in combination with other materials to achieve superior mechanical and thermal properties. They can also be combined with other technologies and materials to develop more sustainable and environmentally friendly products [39].

Biodegradable polymers have been the subject of extensive research [13,38,39,40] and are used in a wide range of applications. Biodegradable plastics have shown promising results in tests, indicating that they can become a viable alternative to non-biodegradable plastics [9]. They are primarily used in packaging, but also in the production of films, fibers, protective coatings for paper and textiles, medical applications such as surgical sutures (e.g., polylactic acid), implants, matrices for controlled drug delivery systems or other active substances, and even in the production of industrial components [41].

Furthermore, the development of biodegradable polymer blends is an important multidisciplinary research direction, closely related to fundamental research in thermodynamics, polymer compatibility, environmental engineering, and biotechnology [42]. Currently, special attention is given to the research and development of new biodegradable materials to reduce environmental impact and meet the growing demand for sustainable and eco-friendly solutions [43]. The gradual replacement of conventional polymer matrix composites with biopolymer matrix composites could reduce CO_2_ emissions by over 85% [44]. Therefore, it is easy to understand the global importance of accelerating studies in the field of biomaterials and biocomposites. Over time, researchers have discovered several possible configurations for composite materials with a polymer matrix. The general conclusion is that biodegradable polymers tend to be formed from ether, ester, or amide bonds [42,44,45].

Some examples of biodegradable polymers that can be used to obtain composite material configurations are biodegradable polyesters (e.g., PLA, PHB, PBS), natural polymers (e.g., cellulose, natural rubber), and general-purpose polymers with biological content (e.g., bio-PE, bio-PP, bio-PET, bio-PC) [46]. Many of these thermoplastic biopolymers are derived from starch and glucose fermentation. Options for thermosetting matrices include common matrices with biological content from natural oils and bioethanol (e.g., bio-epoxy, bio-polyester, bio-polyurethanes) [43,47,48].

Despite the proposed sustainable solutions, previous studies indicate that replacing polymer matrix composites will be a major challenge due to differences in mechanical, physical, and chemical properties [39,42]. Specifically, the articles [44,49] point out that the main issues to be addressed for wider adoption of biopolymers are brittleness, weak chemical resistance, degradation under high temperatures, and temperature resistance. Of course, another significant current disadvantage of biopolymer matrix composites is their cost, which tends to be higher than conventional composites with petrochemical polymer matrices [4,42,49]. Among all the materials mentioned earlier, the most cost-effective and promising in terms of ecological sustainability appears to be polylactic acid (PLA), as it has been demonstrated in the article by [44] that changing the material of an air filter housing from a PA6-GF30 composite to a glass-fiber-reinforced PLA reduces pollutant emissions by 90%.

In addition to polylactic acid (PLA), starch-based plastics have a high potential for replacing non-biodegradable plastics. In a recent study [49], this biodegradable material showed similar strength and elasticity to conventional plastics, indicating solid mechanical strength and the ability to withstand external forces. However, the density of the biodegradable material is higher than that of conventional plastics, which can lead to increased rigidity. Nevertheless, with the development of technological production equipment, it will be possible to achieve lower material density by optimizing the polymerization process. The biodegradable material can be used in a variety of products, including bags, packaging, food containers, and other plastic products. The same study [49] demonstrated that this material has a high melting point (approximately 190 °C), making it more suitable for use in products that need to withstand high temperatures. Another important aspect of this biodegradable material is its high biodegradability, as it degraded by nearly 23% in just 10 days in the tests conducted [9].

Based on the analysis of the results presented in the study by [49], it can be concluded that replacing the polymer matrix derived from the petrochemical industry with a polymer matrix based on biopolymers can be one of the technical solutions to obtain a composite that adheres to eco-design principles. For example, biodegradable materials such as potato-starch-based plastic (TPS-P), corn-starch-based plastic (TPS-C), and polylactic acid (PLA) may exhibit larger dimensional and geometric deviations compared to low-density polyethylene (LDPE), suggesting lower process stability and increased susceptibility to interference from PLA and starch-based materials [49].

The same study [49] also highlights a recurring aspect in the specialized literature: the production of parts from biodegradable polymer materials can achieve an increase in production process productivity of up to 200% compared to LDPE (low-density polyethylene). The biodegradable polymer material that most closely resembles LDPE is TPS-C, while PLA stands out with one of the best fluidities.

Moreover, in additive manufacturing, composite materials with a polymer matrix can be used, representing one of the innovations of this type of manufacturing. This technology allows the creation of three-dimensional objects by successive layering of composite materials, which can be biodegradable [50].

The use of biodegradable composite materials in the 3D printing process offers multiple advantages. Besides being environmentally friendly and sustainable, these materials can be used in various applications, from the production of unique or prototype biodegradable packaging and medical devices to components used in the automotive and aerospace industries [51].

Furthermore, these biodegradable composite materials offer reasonable mechanical and thermal properties, making them suitable for a wide range of applications. For example, in the field of regenerative medicine, these materials can be used to produce gradually degrading artificial tissues and organs within the human body [50,51]. Another benefit of using biodegradable composite materials in 3D printing technologies is that they contribute to reducing the negative impact on the environment. Since the materials are biodegradable, the printed objects can be recycled or naturally decompose, thus reducing waste and pollution [51].

Highly renewable materials available for 3D printing include biodegradable polymer matrices and bio-based fillers, such as fibers or particles, with filler content ranging from a few percent to 40% by volume. Among the most used thermoplastics is PLA, and the reinforcement can consist of cellulose fibers or other natural fibers such as bamboo, birch, coconut, olive, pine, and willow. There are also high-conductivity filaments made from blends of PLA and graphite flakes, as well as soy-based filaments that enhance PLA performance through antimicrobial properties and reduced brittleness [51].

**Figure 4:**
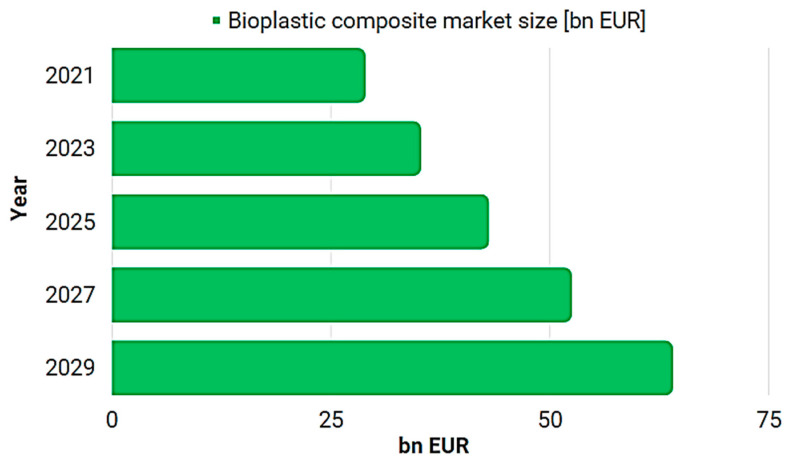
Estimation regarding the evolution of the bioplastic composite materials market.

In the context of increasing concerns about the environment and sustainability, the adoption of composite materials with biopolymeric matrix in various industries is experiencing significant popularity [52]. This trend is the result of the evident advantages that these materials offer compared to synthetic plastic and other traditional options. Composite materials with a biopolymeric matrix, manufactured using renewable raw materials such as cellulose, sugar cane, or natural resources such as corn, represent an ecological and sustainable alternative. Additionally, they exhibit biodegradability and compostability characteristics, being able to decompose in the surrounding environment within a reasonable time frame. These advantages lead to a significant reduction in environmental impact and improvement in the product life cycle. According to the latest research, the adoption of composite materials with biopolymeric matrix is continuously expanding in a wide range of industries, including the food industry, packaging, construction, automobiles, and many others. This sustained growth is highlighted in Figure 4, reflecting the positive trend and progress in the adoption of these innovative materials [52].

The increase in the use of petroleum-based plastics stimulates the demand for bioplastic composite materials. Plastic derived from petroleum poses risks to the environment and involves a costly process based on the extraction and refining of fossil fuels. To counter these issues, bioplastic composite materials are used to reduce dependence on non-renewable biofuel resources in plastic production. An example of such a composite material is starch, which reduces the need for petroleum-based raw materials by 64% [42,52].

The global market for bioplastic composite materials reached a value of $30.92 billion in 2021 and is projected to reach approximately $75.61 billion by 2030, with a compound annual growth rate (CAGR) of 10.44% during the period 2022–2030. This is equivalent to approximately €28.77 billion in 2021 and is estimated to reach approximately €70.61 billion by 2030 [52].

**Q2.2. Polymer composite materials reinforced with natural or hybrid fibers**

The use of natural fiber composites has been increasingly popular in recent times due to their advantages over traditional materials. This type of material is made by incorporating natural fibers into a polymer matrix, creating a hybrid material that combines the strength properties of natural fibers with the flexibility of the polymer matrix [53].

Scientific literature refers to this category of polymeric composite materials as “polymeric green composites”, and it is the most addressed direction in the field of eco-design. Researchers believe that this option is preferred by the industrial factor due to the feasibility of their production process, improved characteristics, and reduced cost. In general, the advantages of these materials include durability, lightweight, low cost, and reduced environmental impact [37,40].

Natural fibers are categorized based on their origin from plants, animals, or minerals. Natural fibers derived from plants can be classified as seed fibers, bast fibers, and leaf fibers. The major chemical components of these fibers are cellulose, hemicellulose, lignin, pectin, and wax. The hydrophilic nature of cellulose affects the interfacial bond between fibers and the usually hydrophobic polymer matrix. Therefore, chemical treatment of fibers can optimize the bond between fibers and the polymer matrix. The most commonly used natural fibers in composite materials include cotton, flax, sisal, jute, and coconut fibers. These fibers are preferred due to their specific properties such as tensile strength, hardness, and rigidity [8,54,55].

**Figure 5:**
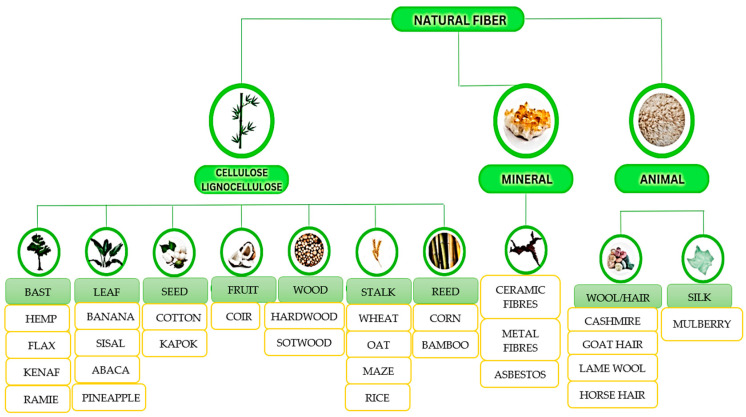
Classification of natural fibers.

By conducting a literature review, we have been able to develop an exhaustive classification of natural fibers based on their origin and their use in composite materials, as shown in Figure 5. We have observed that in most cases, the natural reinforcing fibers used come from various plant sources. In these circumstances, it has been noted that the most commonly used types of natural fibers are derived from corn, bananas, ramie, kenaf, hemp, coir, and others [56].

When referring to their origin in the Animalia kingdom, [36,40,53,55,56] clearly distinguish fibers into two main categories based on taxonomy: animal hair and silk. This taxonomic approach provides additional clarity and allows for a deeper understanding of the origin and characteristics of natural fibers.

The properties of composites significantly depend on the properties of the reinforcing materials, which include natural fibers. The selection of these materials plays a crucial role in determining the performance of durable Fiber-Reinforced Plastics (FRP) composites [53,55,56].

Good performance of composite materials has been achieved when using bast fibers, which are derived from the outer layers of plant stems and primarily composed of cellulose, hemicellulose, and varying proportions of lignin. Fibers obtained from jute, flax, ramie, hemp, and kenaf are frequently used for reinforcing composite materials. Jute is the second most produced natural fiber globally and is biodegradable, recyclable, and eco-friendly. Flax fibers have higher tensile strength than glass fibers, in addition to low density, higher strength, and stiffness. Ramie is one of the oldest plant fibers and is used in the production of fishing nets, ropes, tents, household furniture, and composites. Hemp exhibits excellent mechanical strength and Young’s modulus, as well as good insulation properties, and is commonly used in ropes and mulch. Kenaf fibers are strong, rigid, and tough, with high insect resistance, and have traditionally been used for manufacturing cords, twine, and canvas [40,55].

Thus, it has been observed that natural fibers provide a sustainable alternative to synthetic fibers in composite production. Additionally, the selection of these reinforcing materials is a crucial step to be considered during the design and fabrication of natural fiber composites.

Although natural fiber composites have their advantages, they also present certain disadvantages. For example, natural fibers can be sensitive to water, leading to their degradation over time. Moreover, due to the more complex production process, the manufacturing cost of natural fiber composites can be higher than that of traditional materials [24,57].

However, an increasing number of material manufacturers are starting to utilize natural fiber composites to take advantage of their benefits. These composites are being used in various industries such as automotive, construction, and furniture. According to research, a notable example is the use of natural fibers instead of glass fibers in the development of the trunk frame for the 2017 Mercedes Benz E-Class model. This modification led to a significant weight reduction of 50%. This is a clear demonstration of the impact that alternative materials can have on the performance and efficiency of a vehicle [58].

Therefore, natural fiber composites are a viable alternative to traditional materials, offering specific advantages. However, before being used in production, their disadvantages must be taken into account to ensure the quality and durability of the final product [37,40].

**Q2.3. The sustainability analysis of the two trends in polymer matrix composite materials**

It is important to mention that the sustainability of using a composite material depends on various factors, and the analysis can vary depending on specific applications and contexts [50,59,60]. To conduct a sustainability analysis between composite materials with a polymer matrix and natural or hybrid fiber reinforcement compared to composites with a biodegradable matrix, several key factors can be considered: environmental impact, energy consumption, end-of-life options (recycling, circular economy), performance and durability, water and chemical usage, cost, and market availability.

**The impact on the environment**

- Polymeric Matrix with Natural/Hybrid Reinforcement: Composite materials with a polymeric matrix often use polymers derived from non-renewable fossil fuels, such as petroleum. The extraction and processing of these polymers contribute to greenhouse gas emissions and resource depletion. However, the use of natural or hybrid reinforcements, such as natural fibers (e.g., linen, hemp), can reduce the environmental impact compared to traditional reinforcements made of carbon or glass fibers [24,61].
- Biodegradable Matrix: Composite materials with a biodegradable matrix, such as biologically derived polymers (e.g., polylactic acid, polyhydroxyalkanoates), have the advantage of being derived from renewable sources, reducing dependence on fossil fuels. These matrices can potentially undergo the biodegradation process under appropriate conditions, minimizing the long-term environmental impact. However, the production process and availability of biodegradable matrices may still have some environmental aspects, such as land and water use, depending on the specific material and production methods [62,63].

**Energy Consumption**

- Polymeric Matrix with Natural/Hybrid Reinforcement: The energy consumption required for manufacturing composite materials with a polymeric matrix can be high due to the processing of polymers derived from fossil fuels, including polymerization and blending. These processes are characterized by significant energy consumption, which can result in a larger carbon footprint compared to a composite material with a biodegradable matrix. Moreover, obtaining a ton of natural fibers is considered to consume 4 GJ. In contrast, obtaining glass fibers consumes nearly 8 times more energy, and carbon fibers require 130 GJ/t [64].
- Biodegradable Matrix: Biodegradable matrices often require less energy during the manufacturing process compared to polymers derived from fossil fuels. However, energy consumption can vary depending on the specific material and production techniques [65].

**End-of-Life Disposal, Recycling, Circular Economy**

- Polymeric Matrix with Natural/Hybrid Reinforcement: When considering end-of-life scenarios, composite materials with a polymeric matrix and natural/hybrid reinforcements often face challenges. While natural reinforcements can be biodegradable, the polymeric matrix is generally not, limiting the overall biodegradability of the composite. This can lead to matrix disposal through landfilling or incineration, with certain environmental issues such as waste generation and pollutant emissions. The presence of different materials, such as fibers and polymers, makes separation and recycling more complex. However, efforts are being made to develop efficient recycling processes for these composite materials, aiming to create a circular economy by recovering and reusing materials [66,67,68].
- Biodegradable Matrix: Composite materials with a biodegradable matrix offer the advantage of potential biodegradation at the end of their life cycle. These materials can naturally decompose, reducing the burden on the environment and waste accumulation. However, appropriate disposal conditions, such as specific composting facilities or industrial composting, may be required to facilitate efficient degradation. Biodegradable matrices can provide opportunities for recycling and circular economy approaches. Depending on the specific material, these matrices can be compatible with recycling processes designed for biologically derived polymers. This enables the recovery and reuse of biodegradable composite materials, reducing waste and promoting resource efficiency [20,69,70].

**Performance and Durability**

- Polymeric Matrix with Natural/Hybrid Reinforcement: Composite materials with a polymeric matrix and natural/hybrid reinforcements can exhibit favorable mechanical properties and performance characteristics such as lightweight, strength, and rigidity. However, their durability may be compromised compared to traditional composites with carbon or glass fibers, potentially requiring more frequent replacements [27,71].
- Biodegradable Matrix: Composite materials with a biodegradable matrix may have slightly lower mechanical properties compared to traditional polymeric or natural/hybrid reinforced matrices. However, research and development are ongoing, focusing on improving the performance and durability of biodegradable materials, aiming to reduce performance disparities [49].

**Water and Chemical Usage**

- Polymeric Matrix with Natural/Hybrid Reinforcement: The manufacturing processes involved in composite materials with a polymeric matrix often require large amounts of water and chemicals, such as solvents and resins. The extraction, treatment, and disposal of these substances can increase water requirements and introduce issues related to chemical waste management. Minimizing water consumption and using environmentally friendly chemicals are essential aspects of sustainable production. Additionally, the elimination of chemicals that hinder recycling should be pursued [1,56,72].
- Biodegradable Matrix: Biodegradable matrices can offer advantages in terms of reduced chemical usage. Biologically derived polymers often require less aggressive chemicals during their synthesis and processing compared to polymers derived from fossil fuels. Moreover, some biodegradable matrices can be obtained from secondary agricultural products or waste streams, reducing the need for dedicated agricultural resources and further minimizing environmental impact [63].

**Cost and market availability**

- Polymeric Matrix with Natural/Hybrid Reinforcement: Composite materials with polymeric matrix and natural/hybrid reinforcements can have variable costs depending on factors such as raw material availability, processing techniques, and economies of scale. Natural reinforcements sometimes offer availability advantages compared to traditional carbon or glass fibers, but the overall cost of these composite materials is still higher than that of conventional materials. However, the production cost for natural fibers ranges from $200 to $1000 per ton, for glass fiber it ranges from $1200 to $1800 per ton, and for carbon fiber, the cost is even 10 times higher, reaching up to $12,500 per ton [55,73].
- Biodegradable Matrix: The cost of composite materials with biodegradable matrix can vary depending on factors such as availability and cost of biologically derived polymers, processing techniques, and market demand. In most cases, biodegradable matrices have higher costs compared to traditional fossil-fuel-derived polymers. However, as the demand for sustainable materials increases and mass production develops, cost competitiveness and availability can be exponentially improved [9,52].

Overall, when comparing the sustainability of composite materials with polymeric matrix and natural/hybrid reinforcements versus composite materials with biodegradable matrix, there are specific advantages and challenges for each material type. Therefore, it is important to comprehensively evaluate all these factors and consider the specific application and requirements of the project before making a final decision on using a composite material. The choice should be based on a comprehensive assessment of specific requirements, performance needs, and end-of-life considerations of the intended application, ensuring that the chosen material aligns with overall sustainability goals.

**Q3. When can it be stated that a product made from a composite material with a polymeric matrix meets the requirements of eco-design?**

The analyzed works indicate that eco-design criteria focus on product durability, repairability, recyclability, resource efficiency, and environmental effects, taking into account transportation conditions, industrial structure, and business climate [22,30]. Furthermore, most studies focusing on identifying eco-design criteria highlight that the primary area for improvement in industrial production of parts made from composite materials with a polymeric matrix is sustainability [74].

From a technical perspective, this approach entails the following key considerations when designing a product made from a composite material with a polymeric matrix:

- **Material selection**

It is important that the raw materials used (both the reinforcement and the polymeric matrix) in the manufacturing of the part are responsibly sourced from authorized areas, respecting the rights of the population and ensuring social equity. **Raw material extraction:** The extraction process must comply with ecological standards and environmental protection legislation, using minimal amounts of water and energy to minimize pollutant emissions throughout the extraction process and minimize the impact on extraction sites. An even more demanding criterion is how the extraction area will be restored to its natural state upon completion of the works [41,49].

**Durability:** The chosen materials must be reliable and suitable for the application of the designed and functional parts, possessing the necessary physical, chemical, and mechanical characteristics for the proper use of the final product. Even when considering ecological factors, the design engineer will always choose a material that ensures operational safety and an extended service life, which can be equivalent to the life span of the system in which it is used [56,65,69].

**Ecology:** The materials chosen by the design engineer should be capable of regeneration or easy recycling. These processes should not be polluting or highly water- and energy-consuming, and they should be accessible to small companies as well (in terms of financial sustainability) [46].

**Composite material structure:** The ideal choice is a composite material with a biodegradable polymeric matrix and natural fiber reinforcement. However, since such a composite has limited applications at present and its cost is exponentially higher than that of conventional materials, this criterion will be considered fulfilled if at least one of the following options is chosen: either the polymeric matrix is biodegradable, or the structural reinforcement is of natural origin [64].

**Manufacturing:** The design engineer must choose a material that can be processed using similar technologies, without requiring a significant technological advance or high costs [50].

In conclusion, during the design stage, a material is chosen whose sources do not have a negative and permanent impact on the population and the extraction area. If the material offers suitable physical, chemical, and mechanical properties for the application and can be recycled using standard procedures or if a biodegradable polymer and natural reinforcement are used, then this criterion of eco-design will be considered fulfilled.

- **The manufacturing process**

The manufacturing process should be optimized to reduce energy and natural resource consumption, as well as minimize greenhouse gas emissions and other sources of pollution. It is desirable for the machinery used in the production of composite parts with a polymer matrix to be equipped with the most energy-efficient electric motors, and the energy source should be renewable or partially renewable. Additionally, the manufacturing process should be sustainable and fair to the local workforce [69].

Therefore, if the manufacturing process shows reductions in terms of pollutant emissions, greenhouse gasses, utilizes energy from renewable sources, and has reduced technological water and energy consumption, then this requirement of eco-design can be considered fulfilled [44].

- **Durability**

The product or part produced must be resistant, durable, and safe in operation. It is necessary for the eco-designed part to have a long life span to reduce the need for frequent replacement and premature disposal. Even if biodegradable polymers are chosen for the production of the part, the criterion of durability will not be met if the eco-designed part is not capable of enduring at least as long as the system it is a part of [75,76].

- **Recycling/Regeneration/Repair**

Even though regenerability has become the most important property of polymer-based parts and products, it is currently considered that if a product can be easily recycled, it meets the requirements of eco-design. However, when it comes to composite materials with a polymer matrix, it must be taken into account that polymers generally degrade easily under the high temperatures resulting from mechanical recycling or reintroduction into the injection mold. Additionally, due to the different densities, issues may arise in ensuring uniform reinforcement distribution within the polymer matrix during the injection process. Therefore, an additional step is necessary where the resin and reinforcement are pelletized prior to injection. However, the existing research does not analyze in any way how the reinforcement could be alternatively distributed in the structure of the part, so that mechanically stronger areas contain a higher amount of fiber (either natural or synthetic), or even have reinforced zones while others have no reinforcement at all. We believe that this approach would exponentially increase the percentage of regenerated and recycled materials, especially if it is accompanied by labeling indicating which areas contain only the polymer and which areas contain both the polymer and the reinforcement (the composite material). Considering this opportunity, we believe that this hypothesis should be subject to further research for the advantages it would bring: cheaper parts due to a decreased fiber percentage, reduced energy consumption for fiber manufacturing and transportation, decreased mass of the final product and system, and reduced pollution and water consumption [57,66,77].

It is evident that it is crucial for the product design engineer to consider the possibility of recycling the product after use. However, even more important, in our opinion, is to consider the possibility of repairing the product multiple times until it reaches its end of life. This can be achieved through a design that incorporates multiple modular components that can be easily disassembled and replaced. Creating modular components would facilitate the financial aspect of sustainability by reducing the price of the final product, making the parts more accessible to a larger population. Thus, to meet this requirement, it is desirable for the final product to consist of different assembly organs, instead of solutions that weld or glue two different components together, making it impossible to repair the part.

In conclusion, the recycling criterion can be considered fulfilled from an eco-design perspective if the final part is regenerable, easily recyclable, or repairable using existing technological conditions.

- **Transport**

Carbon emissions and the consumption of non-renewable fossil fuels should be taken into account in multiple stages of the production of composite parts with a polymer matrix. For instance, it is desirable for the factory where the final parts are produced to be as close as possible to the extraction areas of the polymer matrix and reinforcement materials. Such an approach is challenging initially due to limited information available to the design engineer from the manufacturers of each composite component. However, this criterion is more lenient compared to others, but it comes with certain constraints aimed at reducing carbon emissions and energy consumption during transportation. For example, it can be achieved by changing the mode of transportation from road to rail or by replacing the internal combustion engines of transportation machinery with electric or hydrogen-powered engines. Consequently, this criterion will be considered fulfilled if there is a continuous effort, using any means available, to reduce transportation costs, carbon emissions, optimize the quantity of materials transported, and optimize the transportation distance [23,26,74].

## 5. Future Perspectives in the Eco-Design of Polymer Matrix Composite Materials

In the context of future research in the field of eco-design for composite materials with a polymer matrix, there are several key directions that can be addressed:

- Optimizing the structure of composite materials: Investigation can be done to create composite parts with optimized structures, where mechanically stressed areas have a higher percentage of reinforcement, while less functional parts have a lower or even zero amount of reinforcement. This approach could result in parts with similar performance but reduced production costs, logistical advantages, and simplified recycling processes.
- Development of biodegradable composite materials: Another important research direction involves the development of composite materials with a polymer matrix and reinforcements from renewable sources. These materials should offer high performance and operational safety while naturally degrading in the environment, thus contributing to reducing environmental impact.
- Knowledge transfer and best practice examples: Promoting the transfer of knowledge and best practices from the automotive industry and other industrial sectors can be beneficial. By identifying and disseminating efficient eco-design solutions already implemented in various fields, the adoption of these practices can be stimulated in the composite materials industry with a polymer matrix.

These research directions aim to improve the sustainability and efficiency in the use of composite materials with a polymer matrix. By addressing these aspects, it is possible to contribute to reducing the environmental impact and promoting sustainable development in the industry.

## 6. Conclusions

This study focused on the design of polymer matrix composite materials due to the fact that such materials can have variable mechanical, thermal, electrical, and tribological properties. In addition, by applying the principles of eco-design in the case of 3D printing technologies, it allows to increase the number of applications where composite materials with a polymer matrix can be used: biomedicine, the aerospace industry, the military field, the automotive industry, etc. Thus, by applying the principles of eco-design, different techniques can be applied to produce customized substrates in the case of 3D printing. In this scenario, the additive manufacturing (AM) techniques that take into account the principles of eco-design are solutions for obtaining the customized composition that is characterized by a certain morphology of the surface layers.

The implementation of eco-design in all manufacturing phases of composite parts made from polymer matrix materials offers numerous significant advantages from a sustainability and environmental protection perspective. In an increasingly environmentally conscious world, eco-design represents a key concept in developing innovative and sustainable solutions.

One of the main advantages of implementing eco-design in composite materials with a polymer matrix is the reduction of ecological footprint. These advantages include reducing the ecological footprint by using fewer or more durable natural resources, reducing carbon emissions and waste during the manufacturing and usage processes, energy efficiency by effectively utilizing energy throughout the product’s life span, recyclability and biodegradability by choosing polymers and additives that can be recycled or naturally decomposed, durability and longevity of products by developing resilient products with superior mechanical and chemical properties, and reducing the impact on human health by avoiding the use of toxic or harmful substances. All these aspects contribute to environmental and human health protection, as well as efficient resource utilization, making eco-design essential in the development of composite materials with a polymer matrix towards a more sustainable society. Future research in the field of eco-design of polymer matrix composite materials must consider the main aspects related to possible repair or recycling technologies at the end of the life of the products.

## Data Availability

No new data were created or analyzed in this study. Data sharing is not applicable to this article.
